# The Story of the Insane from Year to Year

**Published:** 1902-03-01

**Authors:** 


### THE STORY OF THE INSANE FROM YEAR TO YEAR.

Fourteenth Annual Series.

### CUMBERLAND AND WESTMORLAND ASYLUM.

Here the numbers were practically stationary during the year, there being an increase of 3 only. There were 147 first admissions, 45 readmissions, 77 recoveries, 18 discharged relieved and 72 deaths. he average number resident was 648, and the total number of persons under treatment was 828. There are 60 private patients in residence and 17 out-county cases. Calculated on the admissions the recoveries stand at 40·1 per cent., and calculated on the average number resident the deaths stand 11·1 per cent.; both of these are above the English asylum averages. Of the year's deaths cerebral and spinal diseases account for 36, consumption for 9, pneumonia for 4, and scarlet fever for 1. Of the year's admissions the insanity is attributed to moral causes in 23, to intemperance in drink in 39, to hereditary influence in 52, to previous attacks in 55, and in 30 no cause is assigned. The month of June had the highest number of admissions, namely, 28, and April the lowest, 11. When referring to the admissions Dr. Farquharson says: "Many of the alcoholic cases are ephemeral, the mental symptoms pass off within a few days of admission as soon as the poison is eliminated from the system; these patients invariably promise to give up intemperate habits, but few of them appear to have sufficient self-control to abstain for any length of time." Of course, these cases cannot be kept in the asylums long enough to give them a fair chance. They must be discharged in a few weeks, and they almost invariably return, often three or four times in a year. The committee say "they are of opinion that the efforts of the medical superintendent and the staff of officials to effect the recovery of the patients are very satisfactory." The weekly rate of maintenance is 9s. 0½d. per head.

### EASTERN COUNTIES' ASYLUM FOR IDIOTS.

This asylum contained 254 patients on January 1st, and 255 on December 31st. There were 31 admissions, 15 discharges, and 15 deaths. One of the cases admitted was only 2 years and 9 months old, and another was 41 years, by which it will be seen that this asylum does not place many restrictions on applicants for admission, but that it receives all classes and does its best for them. That much is done we gather from the fact that 51 of the boys are taught trades of various kinds, 16 boys and 19 girls do housework, 10 boys work in the garden, and 34 boys and 22 girls attend school regularly 'a great many others are taught reading, and many of the girls are engaged in needlework. Infectious disease broke out several times in the year, and there was 1 death from diptheria. The average weekly cost of maintenance was 13s. 8½d. per head.

